# Isolation, Characterization and Osteogenic Potential of Mouse Digit Tip Blastema Cells in Comparison with Bone Marrow-Derived Mesenchymal Stem Cells *In Vitro*

**DOI:** 10.22074/cellj.2018.4710

**Published:** 2017-11-04

**Authors:** Leila Taghiyar, Samaneh Hosseini, Mahdi Hesaraki, Forough Azam Sayahpour, Nasser Aghdami, Mohamadreza Baghaban Eslaminejad

**Affiliations:** 1 Department of Stem Cells and Developmental Biology, Cell Science Research Center, Royan Institute for Stem Cell Biology and Technology, ACECR, Tehran, Iran; 2 Department of Developmental Biology, University of Science and Culture, Tehran, Iran

**Keywords:** Blastema Cells, Mesenchymal Stem Cells, Osteogenesis, Regeneration

## Abstract

**Objective:**

Limb regeneration mediated by blastema cells (BlCs) in mammals is limited to the digit tips of neonates. Due to the lack of access to BlCs in adults and the difficulty in isolating and expanding BlCs from neonates, the use of a cellular population with similar features of BlCs would be a valuable strategy to direct a non-regenerative wound towards regeneration. In this study, we have initially isolated and cultured BlCs, and explored their characteristics *in vitro*. Next, we compared the capability of bone marrow-derived mesenchymal stem cells (BM-MSCs) as an alternative accessible cell source to BlCs for regeneration of appendages.

**Materials and Methods:**

In this experimental study, BM-MSCs were isolated from BM and we obtained BlCs from the neonatal regenerating digit tip of C57B/6 mice. The cells were characterized for expressions of cell surface markers by flow cytometry. Quantitative-reverse transcription polymerase chain reaction (qRT-PCR) and lineage-specific staining were used to assess their ability to differentiate into skeletal cell lineages. The colony forming ability, proliferation, alkaline phosphatase (ALP) activity, calcium content, and osteogenic gene expression were evaluated in both BM- MSCs and BlCs cultures at days 7, 14, and 21.

**Results:**

qRT-PCR analysis revealed that the cells from both sources readily differentiated into mesodermal lineages. There was significantly higher colony forming ability in BM-MSCs compared to BlCs (P<0.05). Alizarin red staining (ARS), calcium, and the ALP assay showed the same degree of mineral deposition in both BlCs and BM-MSCs. Gene expression levels of osteblastic markers indicated similar bone differentiation capacity for both BlCs and BM-MSCs at all time-points.

**Conclusion:**

Characteristics of BlCs *in vitro* appear to be similar to BM-MSCs. Therefore, they could be considered as a substitute for BlCs for a regenerative approach with potential use in future clinical settings for regenerating human appendages.

## Introduction

Limb regeneration is a highly complicated dynamic process that differs greatly among various organisms (1, 2). Amphibians such as newts and salamanders have the ability to form completely patterned limbs at any level after amputation (3, 4). However, in mammals such as humans and mice, the regeneration potency of limbs is restricted to the distal region of the terminal phalanx and in neonates (5, 6). Numerous efforts have been made to determine powerful regenerating capability of amphibian species against limited regenerative capacity of adult mammals. Understanding the differences and similarities of wound healing between amphibians and mammalians would provide the ability to manipulate a non-regenerative wound towards regeneration (7).

Limb regeneration consists of three distinct phases that normally commence with the formation of the regenerative epithelium across the plane of amputation. Soon after completion of wound closure, a population of mesenchymal cells [blastema cells (BlCs)] accumulate at the wound site. BlCs ultimately give rise to the musculoskeletal and connective tissues that form the regenerated structure (8, 9). BlCs are located in a limited area associated with the nail organ, and proximal amputation leads to the removal of the nail bed and BlCs (10). BlCs are believed to be a type of stem cell that possesses an undifferentiated state. They originate from either stem cells or dedifferentiation of the mature cells that present in the stump tissue (11). The main surface markers assigned to BlCs are stem cell antigen-1 (Sca-1), endothelial marker (CD31), and vimentin (Vim) (12). It has conclusively been shown that the absence of BlCs and its related genes, which include *Msx1* and *Msx2*, result in the failure of a proximal amputation regeneration in adult mice (13). BlCs enable the process of bone formation to occur by triggering a cascade of the cell signaling pathway that includes bone morphogenetic proteins (BMPs) and fibroblast growth factors (FGFs) (6, 14, 15). Bone formation is considered a main process of limb regeneration only observed in amphibians and neonatal mammals (6). Since the formation of the blastema has been correlated to successful limb regeneration, transplantation of these cells at the amputation site could accelerate wound healing. Nevertheless, the availability of BlCs is a challenging issue. Replacement of BlCs by an available cell source such as mesenchymal stem cells (MSCs) that have the same characteristics could be a valuable strategy in limb regeneration.

MSCs are multipotent cells that exist in most adult tissues, including bone marrow, muscle, and adipose tissues (16-18). They have ability to differentiate into multiple tissue-forming cell lineages (i.e., osteoblasts, adipocytes, chondrocytes, tenocytes, and myocytes) (19-21). Additionally, MSCs preserve their self-renewal capacity following ex vivo expansion (22, 23). It has been postulated that biologically active molecules released by MSCs through paracrine signaling have a reparative effect that consequently affects cell migration, proliferation, and survival of the surrounding cells (24). This paracrine signaling of MSCs also provides anti-scarring properties by the release of hepatocyte growth factor (HGF) and vascular endothelial growth factor (VEGF), and maintenance of the balance between transforming growth factor beta-1 (TGFβ-1) and TGFβ-3 (25, 26). MSCs may also regulate immune and inflammatory responses, and provide therapeutic capability to treat inflammatory diseases (27). It has been hypothesized that a weak inflammatory response caused by a simpler adaptive immune system may result in the higher regenerative capacity in urodele (28, 29). Hence, MSCs would be the best candidate to support successful healing.

Bone marrow-derived MSCs (BM-MSCs), as the gold standard cell source, have been widely investigated for their capacity to regenerate various tissues as well as wound healing properties in over 350 clinical trials worldwide (30). Recently, transplantation of BM-MSCs into an amputated neonate digit tip resulted in increased bone formation (31). Although much research has been devoted to the application and impact of BM-MSCs on bone formation, there has been little investigation to elucidate the potential for MSCs in limb regeneration. Therefore, this study first aimed to isolate, culture and examine the characteristics of BlCs in vitro. Next, we aimed to compare the capability of BM-MSCs as an alternative cell source to BlCs for digit tip regeneration. Herein, BlCs were isolated from neonatal digit tip for the first time and characterized on the basis of morphology, trilineage differentiation capacity, and cell surface markers in comparison with BM-MSCs. Subsequently, we assessed the bone formation ability of both isolated cells by alkaline phosphatase (ALP) activity, expression level of osteoblastic markers, calcium content, and alizarin red staining (ARS). It is believed that BM-MSCs could be utilized as an appropriate cell source for regeneration of proximal digit tip amputation and accelerate wound regeneration through a high innate bone differentiation potential.

C57BL/6 mice (Royan Institute Animal Laboratory) for all of the experiments. BM-MSCs were isolated from mouse BM according to a previously described protocol (32). Briefly, the mice were euthanized in CO_2_ euthanasia chambers. Their tibias and femurs were dissected and cleaned of all surrounding soft tissues. The marrow was slowly flushed out of the bones and suspended in Dulbecco’s modified Eagle’s medium (DMEM, Invitrogen, USA) supplemented with 15% fetal calf serum (FCS, Gibco, Germany), 100 U/ml penicillin (Sigma, Germany), and 100 mg/ml streptomycin. Mononuclear cell fractions were isolated by gradient density centrifugation, plated in a 25 cm^2^ culture flask, and incubated at 37˚C in a humidified atmosphere of 5% CO_2_ for 3 weeks. The cells were subsequently expanded through several passages and we used passage-4 cells for further experiments.

### Isolation and culture of blastema cells

Neonatal (3 day old) C57BL/6 mice were anesthetized with IP administration of 80 mg/kg ketamine (Rotexmedica, Germany) and 8 mg/kg xylazine (Alfasan, Holland). The second and fourth digits of the forelimbs were selected for amputation. The regenerating digits were collected between 7 and 10 days post-amputation (DPA), and digested overnight with 0.2% collagenase type I and 0.5% dispase. Isolated cells were cultured in 24-well tissue culture plates. After 2-3 passages, the adherent cells were used for subsequent analysis. All experimental procedures that involved animals were performed in accordance with the standard operating procedures approved by the Institutional Animal Care and Ethics Committee of Royan institute. Briefly, the mice were kept in cages on a 12-hour light/12-hour dark cycle at 24˚C, and had access to food and water ad libitum. Mice were euthanized by CO_2_ inhalation before BM isolation.

### Flow cytometry

We used flow cytometry to analyze the expressions of cell surface markers for BM-MSCs and BlCs. Passaged-2 BMMSCs and BlCs were trypsinized, washed, and suspended in phosphate-buffered saline (PBS), then incubated with phycoerythrin (PE)-conjugated anti-mouse CD105, CD44 (Becton Dickinson, eBioscience, USA), and fluorescein isothiocyanate (FITC)-conjugated anti-mouse CD90, CD73, 34/45 (Abcam, Becton Dickinson, USA) for 1 hour at 4˚C. Specific antibodies that included PE-conjugated anti-mouse Sca1, CD31 (Abcam, USA), and FITC-conjugated antimouse Vim (Sigma, Germany) were also used for BlCs. The isotype controls consisted of murine FITC-conjugated IgG1 and PE-conjugated IgG2b (eBioscience, USA) as substitutes for the primary antibodies. Data from all samples were collected with a FACScan flow cytometer (BD FACS Caliber, BD Biosciences, San Jose CA USA) and analyzed by Flowing software version 2.5.

### Bone marrow-derived mesenchymal stem cell and blastema cell differentiation to a mesodermal lineage

BM-MSCs and BlCs were evaluated for their ability to differentiate to mesodermal lineages osteoblasts, adipocytes, and chondrocytes. Both cultured BM-MSCs and BlCs were trypsinized and seeded in 6-well culture plates. Osteogenic differentiation was induced by incubating the cells in osteogenic culture medium (DMEM supplemented with 10% FBS, 10 mM β- glycerophosphate, 0.2 mM ascorbic acid, and 1 nM dexamethasone, Gibco, Germanty) for 3 weeks. Osteogenesis was examined by 1% ARS (Sigma, Germanty). We compared the osteogenic capacity of the isolated cells. Differentiation of both BM-MSCs and BlCs to osteoblasts was also assessed at different time points (7, 14, and 21 days). For adipogenic differentiation, we replaced the culture media with adipogenic induction medium [DMEM with 10% FBS, 0.5 mM indomethacin (Sigma, Germanty), l mM ascorbic acid (Sigma, Germanty), and 1 μM dexamethasone (Sigma, Germanty)] for 3 weeks. Lipid droplets in the cells were visualized by 0.4% oil red O staining solution (Sigma, Germanty). A micro-mass culture system was used to induce chondrogenic differentiation of BM-MSCs and BlCs as previously described (32). Briefly, approximately 2.5×10^5^ passage-3 BM-MSCs and BlCs were centrifuged at 1200 g for 5 minutes. The cell pellets were cultured in chondrogenic medium (Lunza, Switzerland) for 21 days at 37˚C and 5% CO_2_ with twice weekly medium changes. Chondrogenic differentiation was assessed by toluidine blue staining of the pellet sections.

### Quantitative reverse transcription polymerase chain reaction measurement

The expression levels of osteogenic, adipogenic, and chondrogenic as well as BlCs related genes were evaluated by qRT-PCR. Total RNA was extracted from cells by using TRI Reagent® (Sigma, Germany). cDNA was produced by the RevertAid First Strand cDNA Synthesis Kit (Fermantas, USA) according to the manufacturer’s instructions. Duplicate qRT-PCR reactions were performed with the SYBR Green Master Mix (Applied Biosystems Life Technologies, Inc., ref: 4367659) with a real-time PCR system (Applied Biosystems Life Technologies, Inc., ABi StepOnePlus) and analyzed with Step One software (Applied Biosystems, version 2.1). The samples were collected from three independent biological replicates. The expression level of target genes was normalized to GAPDH as a reference gene. Analysis was performed by the comparative ΔΔCT method. Table 1 lists the primers.

### Immunocytochemistry

We used immunofluorescence to assess the presence of *Msx1* and *Msx2* as main markers of regeneration as well as BMP4 and FGF8 as bone differentiation and proliferation markers. BM-MSCs and BlCs were fixed in 4% paraformaldehyde (Merck, USA) for 20 minutes and permeabilized with 1% Triton X-100 (Merck, USA). The fixed cells were blocked with 1% bovine serum albumin (BSA, Sigma, Germany) in PBS for 30 minutes at room temperature, then incubated with primary antibodies that included rat polyclonal anti-mouse *BMP4, FGF8, Msx1* and *Msx2* (1:200, Invitrogen, USA) overnight at 4˚C. Cells were subsequently incubated with goat anti-rat Alexa Fluor® 488 secondary antibody (1:500, Invitrogen, USA), and goat antirat Alexa Fluor® 568 secondary antibody (1:500, Invitrogen, USA) for 60 minutes at room temperature. Nuclei were counterstained with DAPI (Invitrogen, USA), followed by a rinse with PBS and subsequently analysis by fluorescence microscope (Olympus BX51, Japan).

### Proliferation and colony-forming unit fibroblasts assay

Cell proliferation was performed using the 3-(4, 5-dimethylthiazol-2-yl)-2, 5-diphenyltetrazolium bromide (MTT) assay. BM-MSCs and BlCs were seeded at a density of 5×10^4^ cells/ml in triplicate in 96-well tissue culture plates. After 1, 3, and 7 days, we added the MTT solution (5 mg/ml) to each well and incubated the plates for 3 hours. Formazan crystals were dissolved in dimethyl sulfoxide (DMSO) and the intensity of the MTT product was measured at 570 nm by a Thermo Scientific™ Multiskan™ GO Microplate Spectrophotometer (Thermo Scientific™, USA). We performed the colony-forming unit fibroblast (CFU-F) assay to the evaluate proliferation potential of the isolated cells. Approximately 1000 passage-1 cells were plated in 60-mm dishes and allowed to proliferate for one week. The cultures were then fixed and stained by crystal violet for 10 minutes. Colonies were counted under an invert phase contrast microscope (Olympus, USA).

### Alkaline phosphatase activity

The differentiation of both BM-MSCs and BlCs to osteoblast cells was evaluated as a function of ALP activity after 7, 14, and 21 days. ALP activity was assessed using an Alkaline Phosphatase Assay Kit (Colorimetric, Abcam, USA, ab83369) according to the manufacturer’s protocol. Briefly, cells were grown on 6-well plates at a density of 2×10^5^ cells per well. The medium was replaced after 72 hours by 0.2 mM ascorbic acid, 10 mM β-glycerophosphate, and 1 nM dexamethasone that contained growth medium. The cell layers were washed with PBS and scraped off from the plates’ surfaces by lysis buffer. After sonication and centrifugation, aliquots of the cell lysis solution were collected for analysis of ALP activity and total protein content. ALP activity was determined with respect to the release of p-nitrophenol from p-nitrophenyl phosphate substrate. Each reaction was initiated by the addition of p-nitrophenyl phosphate to the cell lysis solution and stopped after 60 minutes by the addition of a stop solution. Optical density was measured at 405 nm using a Thermo Scientific™ Multiskan™ GO Microplate Spectrophotometer (Thermo Scientific™, USA). ALP activity values were normalized with respect to the total protein content obtained from the same cell lysate and expressed as units per microgram of total proteins. Total protein content was determined using the BCA protein assay kit (EMD Millipore Co., Darmstadt, Germany). The absorbance of the reaction product was measured at 562 nm. The protein concentration was calculated from a standard curve.

**Table 1:** Description of mouse primers used in quantitative-reverse transcription polymerase chain reaction


Gene symbol	Primer sequencing (5ˊ-3ˊ)	Accession number	Annealing time (°C)

*Gadph*	F: ACTTCAACAGCAACTCCCAC	NM_008084	60
	R: TCCACCACCCTGTTGCTGTA		
*Fgf8*	F: GGGGAAGCTAATTGCCAAGA	NM_001166361.1	60
	R: CCTTGCGGGTAAAGGCCAT		
*Bmp4*	F: GTCGTTTTATTATGCCAAGTC	NM_001316360.1	60
	R: ATGCTGCTGAGGTTGAAGAG		
*Msx1*	F: CTGCTATGACTTCTTTGCC	NM_010835.2	60
	R: CTTCCTGTGATCGGCCAT		
*Msx2*	F: CACCACATCCCAGCTTCTA	NM_013601.2	60
	R: GCAGTCTTTTCGCCTTAGC		
*Runx2 *	F: CAGCATCCTATCAGTTCCCAA	NM_001145920.2	60
	R: CAGCGTCAACACCATCATT		
*Col Ia1*	F: CAAGAAGACATCCCTGAAGTC	NM_007742.4	60
	R: ACAGTCCAGTTCTTCATTGC		
*Ocn*	F: AAGCAGGAGGGCAATAAGGT	NM_001032298.3	60
	R: CAGAGTTTGGCTTTAGGGCA		
*Alpl*	F: GCCAGCAGGTTTCTCTCTTG	NM_001287172.1	60
	R: GGGATGGAGGAGAGAAGGTC		
*Pparg*	F: GAGCACTTCACAAGAAATTACC	NM_011146.3	60
	R: AATGCTGGAGAAATCAACTG		
*LpL*	F: AATGCCATGACAAGTCTCTG	NM_008509.2	60
	R: AAACCCACTTTCAAACACCC		
*Adiponectin*	F: TGTTCCTCTTAATCCTGCCCA	NM_009605.4	60
	R: CCAACCTGCACAAGTTCCCTT		
*Col II*	F: ATGATCCGCCTCGGGGCTC	NM_001113515	60
	R: GGGCCTGTCTGCTTCTTGTA		
*Sox9*	F: TGAATCTCCGGACCCCTTCATG	NM_011448.4	60
	R: CACAGCTCACCAGACCCTGAG		
*Aggrecan*	F: ATGACCACTTTACTCTT	NM_007424.2	60
	R: CCCAGCATGGCCCACTGA		


### Calcium assay

The ability of the isolated cells to produce mineralized matrix was assessed by measurement of calcium content at days 7, 14, and 21 post-induction. The calcium concentration was measured using a Calcium Colorimetric Assay Kit (Biovision, Inc., USA), which is based on the formation of stable purple colored complexes that are particularly visualized with free calcium. Color intensity was measured at 575 nm using Thermo Scientific™ Multiskan™ GO Microplate Spectrophotometer (Thermo Scientific™, USA) and is directly proportional to the calcium concentration of the samples.

### Statistical analysis

Statistical analyses were carried out on datasets that consisted of at least three independent experiments using an unpaired student’s t test when comparing two groups. We used one-way ANOVA with Tukey’s multiple comparison test when comparing more than two groups or two-way ANOVA with Tukey’s multiple comparison test for nonparametric results with GraphPad Prism software (GraphPad, San Diego, CA, USA). All data are expressed as mean ± SD. *P<0.05, **P<0.01, ***P<0.001, and ****P<0.0001 defined statistical significance.

## Results

### Characteristics, morphology, and cell surface markers of bone marrow-derived mesenchymal stem cells and blastema cells

**Fig.1:**
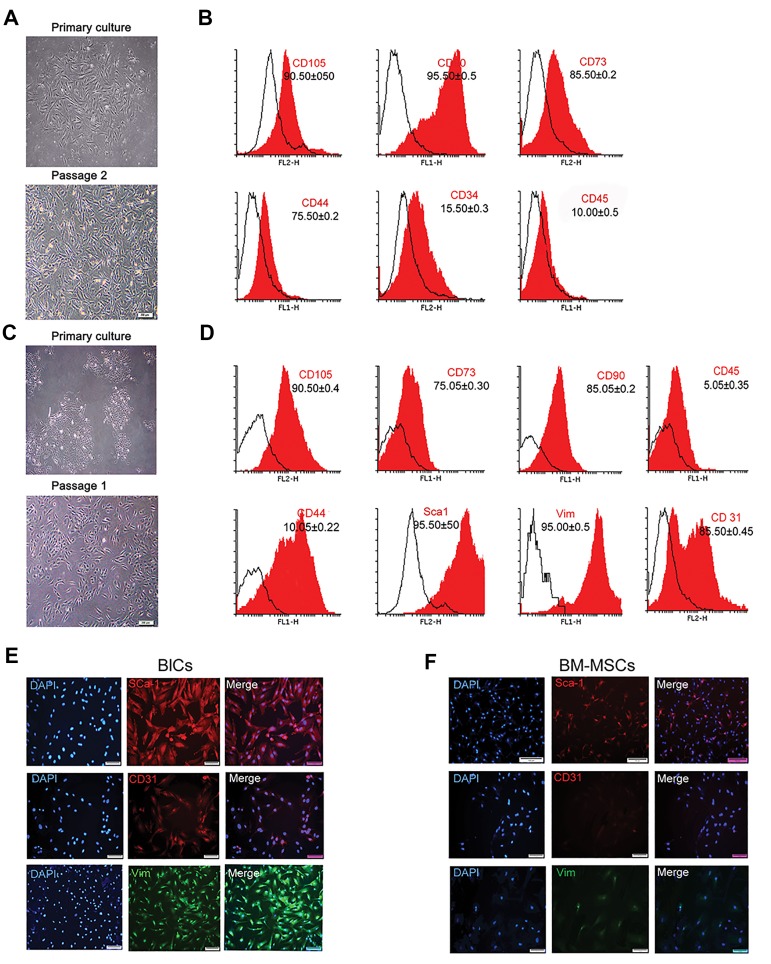
Characterization of bone marrow-derived mesenchymal stem cells (BM-MSCs) and blastema cells (BlCs). A. Primary and passage 2 culture of BM-MSCs, B. Flow cytometry analysis of cell surface marker expressions for BM-MSCs, C. Primary and passage-2 culture of BlCs, D. Expression of cell surface markers for BlCs, E. Immunofluorescence staining of BlCs, and F. BM-MSCs with anti-vimentin (Vim), Sca1, and CD31 showed that BlCs had higher expression levels of these markers.

We isolated and expanded plastic-adherent cells that had a typical fibroblastic-like shape from both donor tissues (Fig .1A, B). BlCs had a slightly smaller size compared to BM-MSCs. The first colonies from BM-MSCs appeared within 3 to 5 days after plating, while BlCs began to adhere to the bottom of the dish and form discrete colonies 7 to 10 days after plating. We used flow cytometry analysis to confirm the stem cell phenotype of the isolated BMMSCs and BlCs by assessing cells from each group against various surface markers (CD90, CD105, CD73, CD 44, CD34, and CD45). As expected, the majority of BM-MSCs tested positive for CD90, CD105 (>90%), CD73 (85%), and CD44 (75%). In addition, 10% of BMMSCs expressed CD34, whereas 15% expressed CD45 (Fig .1C). As shown in Figure 1D, more than 75% of BlCs expressed CD90, CD73, and CD105. However, BlCs did not express for CD44 since it was expressed in less than 10% of the cells. As much as 85% expressed Sca- 1, 80% expressed CD31, and 60% of the BlC population expressed Vim (Fig .1D). Immunofluorescent analyses that used anti-Sca-l, CD31, and Vim antibodies showed higher expressions of these specific markers in BlCs compared to BM-MSCs (Fig .1E, F).

**Fig.2:**
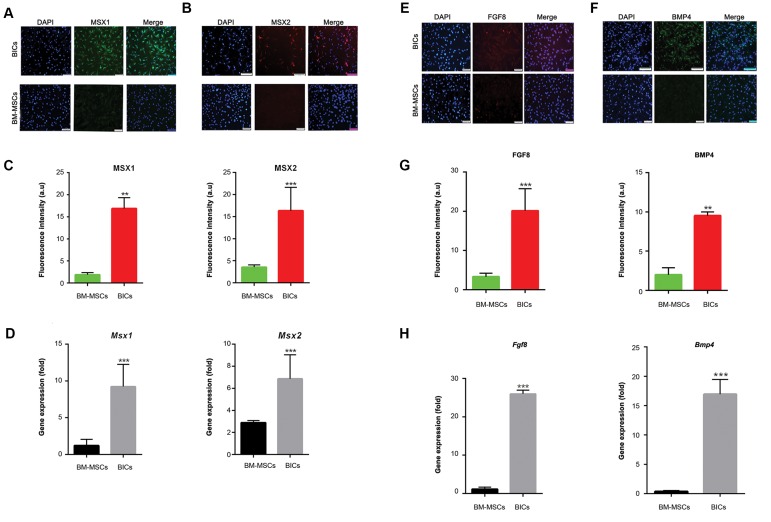
Expression level of *Msxs, Bmp4,* and *Fgf8* genes, and their related proteins. Immunofluorescence staining of A. *Msx1*, B. *Msx2*, and their related fluorescent intensity, C. In both blastema cells (BlCs) and bone marrow-derived mesenchymal stem cells (BM-MSCs). *Msx1* (green), *Msx2* (red) and nuclei (DAPI, blue). Right panel shows merged image with DAPI, D. Gene expression levels of *Msx1* and *Msx2* in BlCs and BM-MSCs. Immunofluorescence staining for E. FGF8 (red) and F. BMP4 (green), G. As well as their related fluorescent intensity in BlCs and BM-MSCs, and H. Histogram shows the expression levels of *FGF8* and *BMP4* in BlCs and BM-MSCs [scale bar: 100, means ± SD (n=3)]. **; P<0.01 and ***; P<0.05.

Protein expression levels of *Msx1* and *Msx2* were evaluated by immunofluorescence. The expression levels of *Msx1* (green) and *Msx2* (red) dramatically increased in BlCs compared to BM-MSCs (Fig.2A, B). The percentage of MSX positive cells was approximately 20 ± 5% for BlCs and less than 3 ± 2% for BM-MSCs (Fig .2C). qRT-PCR analysis indicated that the *Msx1* and *Msx2* genes upregulated by 10-12 fold in BlCs (Fig .2D). BMP4 (green) and FGF8 (red) proteins significantly expressed in BlCs, but were slightly detected in BM-MSCs (Fig .2E, F). BMP4 protein expressed in 25% of BlCs and 5% of BMMSCs. Fgf8 expressed in 10% of BlCs and 3% of BMMSCs (Fig .2G). Analysis of *Fgf8* and *Bmp4* showed a statistically significant higher gene expression levels in BlCs compared to BM-MSCs (Fig .2H, ***P<0.01).

### Differentiation potential of bone marrow-derived mesenchymal stem cells and blastema cells into mesenchymal lineages

**Fig.3:**
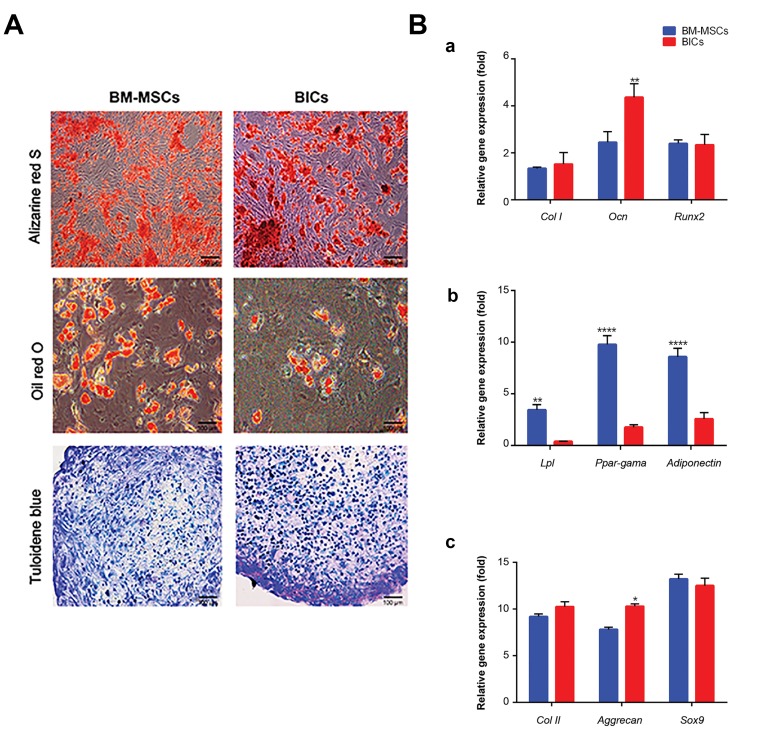
Differentiation potential of bone marrow-derived mesenchymal stem cells (BM-MSCs) and blastema cells (BlCs) into mesenchymal lineages. A. The images represent the differentiation potential of BMMSCs and BlCs to osteoblasts, adipocytes, and chondrocytes following alizarin red-S, oil red-O and toluidine blue staining, respectively and B. Quantitative-reverse transcription polymerase chain reaction (qRT-PCR) data for (a) osteoblastic (*Col I, Runx2, Ocn*), (b) adipogenic (*Ppar-G, Lpl, adiponectin*), and (c) chondrogenic (*Col II, Sox9*, and *aggrecan*) related genes obtained after 21 days for the BlC and BM-MSC groups [mean ± SD (n=3)]. *; P<0.05, **; P<0.01, and ****; P<0.0001.

Differentiation of BM-MSCs and BlCs toward an osteoblastic lineage was assessed by ARS and qRT-PCR. ARS results confirmed the presence of calcium minerals in the extracellular matrix of both BM-MSCs and BlCs. Mineral deposition started at day 7 and increased considerably up to day 21 (Fig .3A). qRT-PCR analysis of osteogenic related genes showed no significant differences in the expression levels of *Col I* and Runx2 between BlCs and BM-MSCs. In contrast, Ocn had a higher gene expression level compared with BM-MSCs (Fig .3Ba). Oil red O staining and qRT-PCR detected adipogenic differentiation of BM-MSCs and BlCs. Intracellular oil droplets accumulated in both BM-MSCs and BlCs. The size and number of oil droplets increased during the 3 weeks of cultivation (Fig .3A). Analysis of adipogenic related genes (*Lpl, Ppar-G*) and adiponectin indicated a higher expression level in BM-MSCs compared to BlCs (Fig .3Bb). The ability of BM-MSCs and BlCs to undergo chondrogenic differentiation was assessed by qRT-PCR analysis of *Col II, aggrecan*, and *Sox9* genes as well as toluidine blue staining. After 21 days, toluidine bluestained areas on the cross-sections indicated the existence of sulfated proteoglycans in both BlCs and BM-MSCs (Fig .3A). Analysis of genes involved in chondrogenesis showed that both BlCs and BM-MSCs expressed comparable levels of *Col II* and *Sox9*. Similarly, there was no considerable difference in the expression level of *aggrecan* among the samples, although it partially upregulated in BlCs (Fig .3Bc).

### Proliferation and colony-forming unit fibroblast assay

**Fig.4:**
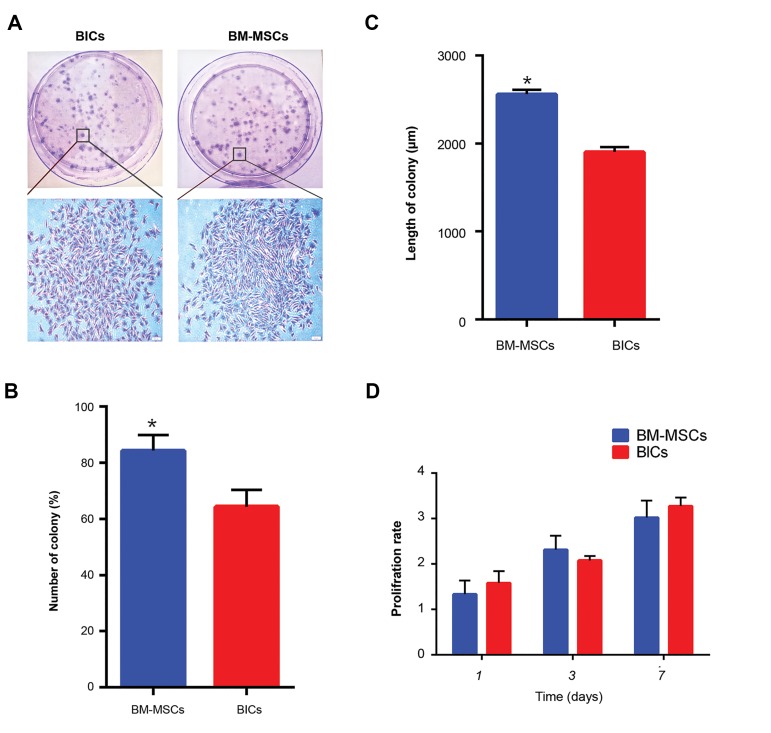
Cell proliferation and colony-forming assay (CFU). A. Bone marrowderived mesenchymal stem cells (BM-MSCs) and blastema cell (BlCs) colonies visualized by crystal violet staining, B. Histogram of the numbers and length of colonies, C. In BM-MSCs and BlCs, shows that BM-MSCs have longer and higher number of colonies, and D. Proliferation of BM-MSCs and BlCs using MTT assay at days 1, 3, and 7 showed a similar proliferation rate between BM-MSCs and BlCs.

We used the CFU-F assay to examine the proliferation pattern of BlCs and BM-MSCs in a semisolid medium (Fig .4A). The colonies from each culture dish and average number of colonies per dish. The results showed 80 ± 5 BM-MSC colonies and 60 ± 5 BlC colonies (Fig .4B). Accordingly, the BM-MSCs colonies were significantly longer compared to the BlCs (Fig .4C). The proliferation of both BlCs and BM-MSCs after 1, 3, and 7 days (Fig .4D). The results indicated that BlCs had greater proliferation compared to BM-MSCs on days 1 and 7, even though this difference was not statistically significant.

### Osteogenic activity of bone marrow-derived mesenchymal stem cells and blastema cells

**Fig.5:**
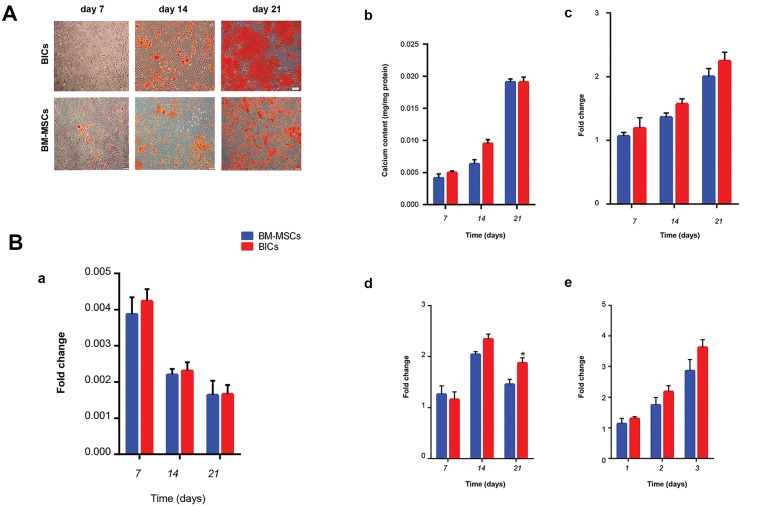
Osteogenic activity. A. Alizarin red staining (ARS) of bone marrow-derived mesenchymal stem cells (BM-MSCs) and blastema cells (BlCs) after 7, 14, and 21 days. Nodule-like aggregations formed within 3 weeks in BlCs and BM-MSCs and B. Quantitative-reverse transcription polymerase chain reaction (qRT-PCR) analyses of (a) alkaline phosphatase (ALP) activity and (b) calcium deposition showed similar osteogenic activity in both BlCs and BM-MSCs. Additionally, (c) *Col I*, (d) *Runx2*, and (e) *Ocn* expressions upregulated similarly in both groups.

We assessed the osteogenic activity of BM-MSCs and BlCs at various time points (culture days 7, 14, 21) using ARS. Nodule-like aggregates began to form during the first week and increased in abundance toward the end of the third week. These mineralized nodules were more significant in the BM-MSCs plates compared to BlCs (Fig .5A). As an early marker for osteogenic differentiation, ALP activity was measured after 7, 14, and 21 days of incubation (Fig .5Ba). After 7 days, both BM-MSCs and BlCs showed an almost equal increase in ALP activity. ALP activity significantly decreased in all studied groups at days 14 and 21. The calcium content after 7, 14, and 21 days of culture for both BM-MSCs and BlCs (Fig .5Bb). The calcium content increased over time in both groups. After 14 days, BM-MSCs had a higher calcium content compared to BlCs. There was a 2-fold increase in calcium content in both BM-MSCs and BlCs after 21 days of culture. We observed no significant differences in calcium content between BM-MSCs and BlCs.

qRT-PCR analysis revealed that the *Col 1* expression progressively upregulated within 3 weeks (Fig .5Bc). However, *Runx2* and *Ocn* significantly expressed within 2 weeks and subsequently downregulated (Fig .5Bd, e). Overall, the fold changes for all genes were comparable between BM-MSCs and BlCs at all of the time points.

## Discussion

Blastema formation is a transient, important step in the limb regeneration process, the absence of which in proximally amputated digit tips of adult humans and mice is a major challenge for functional regeneration (33). The isolation and expansion of BlCs from neonatal digit tips in mice is highly complicated and time-consuming. Hence, an *in vitro* study of BlCs is rare. The use of alternative BlC sources would be promising for digit regeneration. Here, we successfully isolated BlCs and compared their characteristics with BM-MSCs, as a possible substitute for BlCs under cell culture conditions. Cell behavior, differentiation potentials, and the bone formation ability of both BM-MSCs and BlCs were assessed. In this study, the successful isolation of BlCs and BM-MSCs was determined according to their plastic-adherent ability, morphology, and expression of specific cell surface markers (32). Both BlCs and BM-MSCs showed heterogeneous cell populations in primary culture, which became relatively homogeneous after 2-3 passages. The CFU-F assay showed that both groups had the ability to form colonies, even though the BM-MSCs culture had significantly greater size, number, and growth rate of colonies. Numerous factors such as cell proliferation, cell death, cell migration, growth factor secretion, and matrix turnover impact on the size and number of colonies in the CFU assay (34). Therefore, due to the differences between BlCs and BM-MSCs in the above mentioned factors, we expected this discrepancy in size and colony number. The MTT assay showed a similar proliferation rate for BM-MSCs and BlCs, which has confirmed that BM-MSCs would be an appropriate alternative candidate for BlCs due to the proliferation process which is critical for mammalian tissue repair (35).

Isolated stem cells from both sources were also identified on the basis of specific cell surface markers. Analysis of cell surface markers revealed that both cell populations expressed the mesenchymal cell markers CD73, CD90, and CD105. The majority of BM-MSCs were negative for CD34 and CD45, whereas BlCs had negative results for CD44. These results agreed with other studies that characterized BM-MSCs from various sources (36). A number of attempts have been made to determine the origin of BlCs (37). Although the exact origin is not clear, a strong belief exists that BlCs are associated with stem cells by mesenchymal origin (37, 38), which we have observed in this study. In addition, CD31, Vim and Sca-1 are considered specific markers for BlCs. Flow cytometry and immunofluorescence results clearly confirmed elevated expressions of the BlCs-specific markers, which were negligible in BM-MSCs.

We examined the ability of both groups of cells to differentiate into a skeletal lineage. Both isolated cells had a comparable differentiation potential towards chondrocytes and osteoblastic lineages. The ability of BMMSCs to differentiate into mesodermal lineage has been well documented (39). In this study, the chondrogenic and osteogenic ability of the isolated cells were supported by the appearance of proteoglycans and mineralized nodules that stained positively with toluidine blue and alizarin red, respectively. Studies have shown that BlCs have the ability to differentiate into skeletal cells such as bones and cartilage (40, 41). We detected upregulation of osteogenic and chondrogenic related genes for BM-MSCs and BlCs, which has confirmed that they belong to a mesenchymal cell source (16). Lipid accumulation on both BM-MSCs and BlCs confirmed adipogenic differentiation. However, BM-MSCs had a greater adipogenic potential compared with BlCs. Mechanisms that promote one cell fate are believed to actively suppress mechanisms that induce an alternative lineage (42). BM-MSCs can give rise to a range of other cell types due to their multipotency, whilst BlCs, as progenitors, have a pre-determined cell fate. Thus, the shift in BlCs differentiation to an osteoblast lineage, but not adipogenic, may contribute to the activation of BMP signaling and bone formation.

MSXs are considered to be regeneration-specific genes that normally express in BlCs. They regulate BlCs growth, cell differentiation, bone formation, and are essential for the generation of a functional limb (13, 43). The results of qRT-PCR have shown significant changes in the expression levels of *Msx1* and *Msx2* in BlCs and BM-MSCs. Similarly, based on immunofluorescence results, we observed a dramatic increase in *Msx1* and *Msx2* protein levels in BlCs. In agreement with these findings, *Bmp4* and *Fgf8* expression levels significantly upregulated in the BlCs. Evaluation of protein expression by immunofluorescence also confirmed the upregulation of BMP4 and BlCs. BMP plays a key role in bone development and skeletal repair, as well as an endogenous regeneration response (44). FGF8, as one of critical signaling molecules during blastema formation, is connected to initiation, outgrowth, and patterning of vertebrate limbs (14).

In order to elucidate precisely the bone formation ability of BlCs, we evaluated osteoblastic differentiation of BlCs and BM-MSCs by assessments of ALP activity, calcium content, and qRT-PCR at different time-points. ALP, as an early marker for osteoblastic differentiation, significantly increased in both cell types within the first 7 days. We have observed a substantial decrease in ALP at later stage in both BM-MSCs and BlCs. It is known that the early osteogenic differentiation starts with an increase in ALP activity; its level declines when other osteoblastic genes upregulate prior to calcium deposition (45). Our results have clearly represented this typical expression profile of ALP. In accordance with the ALP results, the calcium content significantly increased in BlCs and BM-MSCs when the ALP activity declined. Osteogenic differentiation was confirmed by ARS which preferentially stains the mineralized nodules. Based on the current results, we observed a progressive increase in the amount of mineralized nodules over 3 weeks of osteoinduction for both BM-MSCs and BlCs.

Gene expression analyses of *Runx2, Col I* and *OCN* have also confirmed upregulation of osteogenic related genes in the BlCs and BM-MSCs groups. Runx2, as an early marker of differentiation, is known to activate the expression of osteogenic-related genes, including collagen I, osteopontin, osteocalcin, and bone sialoprotein (46). Accordingly, *Runx2* expression increased at days 7 and 14, and declined at day 21. Col I is one of the first extracellular matrix protein generated during bone induction (47). The higher expression level of Col I has proven the bone formation ability of BM-MSCs and BlCs. *Ocn* is another osteogenic marker that begins to express during the late stage of differentiation (48). Upregulation of *Ocn* at day 21 confirmed that BM-MSCs and BlCs could induce ECM formation in the late stage. Therefore, our qRT-PCR data were consistent with the observed mineral nodules detected by ARS and calcium content which supported the bone formation ability of BM-MSCs and BlCs.

BMP4, as a key factor during bone development, accelerated the process of bone formation. qRT-PCR analysis showed a slightly higher expression level of osteoblastic genes in the BlCs group. Therefore, the elevated osteogenic activity of BlCs relative to BMMSCs might relate to a superior BMP4 gene expression level in BlCs.

## Conclusion

BM-MSCs share similar properties with BlCs such as developmental potency, proliferation and, more importantly, modulation of immune and inflammatory responses. MSCs, in addition to providing an accessible cell source, can accelerate wound regeneration through high innate bone differentiation potential. Hence, they can be used as an appropriate cell source in prospective therapeutic applications for limb regeneration. However, additional studies in animal models that use genetically modified or engineered MSCs could provide a better understanding of the regeneration process in proximal digit tip amputation.
